# A Comprehensive Review of Biological Agents for Lupus: Beyond Single Target

**DOI:** 10.3389/fimmu.2020.539797

**Published:** 2020-10-02

**Authors:** Bingyi Yang, Ming Zhao, Haijing Wu, Qianjin Lu

**Affiliations:** Department of Dermatology, The Second Xiangya Hospital of Central South University; Hunan Key Laboratory of Medical Epigenomics, The Second Xiangya Hospital of Central South University, Changsha, China

**Keywords:** SLE, belimumab, bispecific antibodies, tibulizumab, biological therapy

## Abstract

Systemic lupus erythematosus (SLE) is an autoimmune disease that involves multiple immune cells. Due to its complex pathogenesis, the effectiveness of traditional treatment methods is limited. Many patients have developed resistance to conventional treatment or are not sensitive to steroid and immunosuppressant therapy, and so emerging therapeutic antibodies have become an alternative and have been shown to work well in many patients with moderate and severe SLE. This review summarizes the biological agents that are in the preclinical and clinical trial study of SLE. In addition to the various monoclonal antibodies that have been studied for a long time, such as belimumab and rituximab, we focused on another treatment for SLE, bispecific antibodies (BsAbs) such as tibulizumab, which simultaneously targets multiple pathogenic cytokines or pathways. Although the application of BsAbs in cancer has been intensively studied, their application in autoimmune diseases is still in the infant stage. This unique combined mechanism of action may provide a novel therapeutic strategy for SLE.

## Introduction

Systemic lupus erythematosus (SLE) is a heterogeneous autoimmune disease, and the pathogenesis involves genetic factors, epigenetics, environmental factors, which resulting in immune abnormalities. Immune abnormalities are mainly the loss of tolerance and sustained autoantibody production (1). The main immunological manifestations are the abnormal activation of T cells and B cells with abundant autoantibodies that form antigen-antibody complexes in tissues and organs, which results in damage and inflammation (2).

**FIGURE 1:**
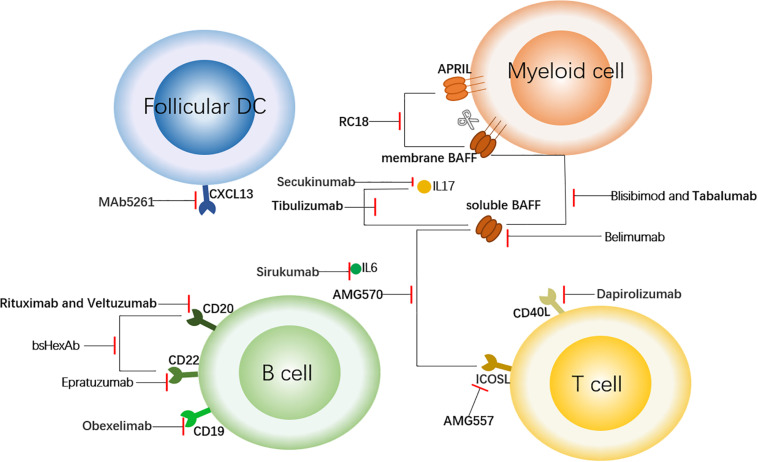
Targeted Therapy of SLE Centered on B Cells. This figure shows the sites of action of some therapeutic antibodies with a focus on B cells. The antibodies shown here bind to the surface molecules of B cells and down-regulate the immune response. In addition, to block the upstream factors regulating B cells (such as BAFF and APRIL) or downstream inflammatory factors such as IL6, so as to achieve the role of regulating immune response. The short red line indicates that the antibody has a blocking effect on the corresponding cell receptor or cytokine. follicular DC, follicular dendritic cell; CXCL13, chemokine ligand 13; APRIL, a proliferation-inducing ligand; BAFF, B cell activation factor; CD40L, CD40 ligand; and ICOSL, inducible T cell co-stimulator ligand.

With a deepening understanding of the pathogenesis, targeted therapy has become a more promising treatment, especially for the patients who not respond to conventional treatments. Conventional treatments, mainly including glucocorticoids and immunosuppressants, have poor specificity and are prone to tolerance. SLE patients have an increase in multiple cytokines and auto-antibodies, and there may be significant differences in cytokine levels in different patients, such as I interferon (IFN) levels (3). This provides strong support for blocking specific cytokines or pathways with specific antibodies. In this review, we will summarize the existing biological agents, expound on their effects at different sites (Figure 1), and hope to shed light on future research to develop more targeted therapy.

## Targeting B Cells

B cells are central to the pathogenesis of SLE. Dysregulation of transcription factors and cytokines in B cells and interaction between B-T cells can lead to abnormal maturation of B cells and the production of autoantibodies (4, 5). Targeted blocking of B-cell-related cytokines has an obvious effect on down-regulating the overly strong immune response.

### BAFF/APRIL Inhibition

B cell activation factor (BAFF, or BLyS), which regulates the survival and maturation of B lymphocytes, is a member of the TNF family and has both a membrane form and soluble form (6). BAFF has been found to play an important role in the survival and differentiation of B cells in recent years. By binding to three different receptors, BAFF-R, TACI and BCMA, BAFF promotes B cell differentiation, maturation and class conversion, promoting the humoral immune response and participating in T cell activation (7, 8). APRIL (a proliferation-inducing ligand) is also a member of the TNF family, has high homology with BAFF, and binds to the receptors TACI and BCMA. Excessive expression of BAFF promotes the malignant proliferation of B cells and leads to autoimmune diseases (9).

Belimumab is a fully humanized IgG1 monoclonal antibody (mAb) that only binds to soluble BAFF and blocks its binding to the three receptors (10), directly reducing naive and transient B cells and indirectly inhibiting the function of IgD-CD27^++^ memory B cells and plasma cells (11). This is the first biological agent to be approved by the FDA for SLE. Early multicenter phase III clinical trials have shown that longterm use of high doses continuously improved serological indicators, reduced hormone dosage and reduced the risk of severe recurrence in SLE (12, 13). Real world study make us more comprehensive understanding of this drug. A retrospective study of 466 patients with active SLE found that the lower the baseline damage, the greater the probability of achieving remission, indicating the benefits of early medication for SLE (14). Currently Belimumab in childhood – onset systemic lupus erythematosus (cSLE) II period in the clinical trials have been successfully developed, and the efficacy is consistent with adults (15) (Table 1).

**TABLE 1:** Single-target biological agents in SLE.

Biologic	Agent type	Mechanism of action	Stage in SLE to date	References
**Targeting B cells**				
Belimumab	Anti-BAFF mAb	Binding to soluble BAFF	On the market	(10–15)
Tabalumab	Anti-BAFF mAb	Binding both soluble and membrane BAFF	Phase III	(16–19)
Blisibimod	Anti-BAFF fusion protein	Binding both soluble and membrane BAFF	Phase III	(20–23)
Epratuzumab	Anti-CD22 mAb	Binding to CD22	Phase III	(32–36)
Rituximab	Anti-CD20 mAb	Binding to CD20	On the market	(37–40)
Veltuzumab	Anti-CD20 mAb	Binding to CD20	Case report	(41, 42)
MAb5261	Anti-CXCL13 mAb	Binding to CXCL13, interfere with the migration of B cells	Preclinical	(46, 47)
**Targeting co-stimulators**				
Dapirolizumab	Anti-CD40L mAb	Binding to CD40L, inhibiting co-stimulation and B cell maturation	Phase II	(50–54)
Abatacept	CTLA4-Fc fusion protein	Interfering with T cell activation	Phase IIb	(56–58)
AMG557	Anti-ICOSL mAb	Binding to ICOSL	Phase II	(59–61)
1D1	Anti-CD86 mAb	Binding to CD86	Preclinical	(64)
**Targeting cytokine**				
Tocilizumab	Anti-IL-6R mAb	Blocks the binding of IL-6 and IL-6R	Phase II	(68–70)
Sirukumab	Anti-IL-6 mAb	Binding to IL-6	Phase II	(71, 72)
Secukinumab	Anti-IL-17A mAb	Binding to IL-17A	Case report	(74, 75)
Sifalimumab	Anti-IFNα mAb	Binding to most subtypes of IFN	Phase IIb	(77–80)
Rontalizumab	Anti-IFNα mAb	Blocking inflammation induced by type I IFN	Phase II	(81, 82)
Anifrolumab	Anti-IFNαR mAb	Blocks the binding of IFNα and IFNαR	Phase III	(83–85)
Infliximab	Anti-TNFα mAb	Neutralizing TNF in peripheral blood	Case report	(87)
Ustekinumab	Anti-p40 mAb	Binding to the IL12/IL23 subunit p40	Phase II	(90, 91)
**Targeting complements**				
Eculizumab	Anti-C5 mAb	Binding to complement C5	Case report	(93–96)

Tabalumab is a humanized IgG4 single-chain antibody that can bind to both membrane and soluble BAFF (16). In randomized phase II trials of rheumatoid arthritis (RA), treatment resulted in transient increases in the total number of B cells, naive B cells, and memory B cells (17). Two phase III studies evaluated the role of tabalumab in patients with moderate to severe SLE. One showed that although tabalumab treatment resulted in significant changes in the biological activity of anti-dsDNA, complement, B cells and immunoglobulin, the primary endpoint was not achieved (18). Another study showed that key secondary endpoints were not met and that side effects were depression and suicidality (19). In response to these results, tabalumab development has been discontinued.

Blisibimod is an antagonistic peptide-FC fusion protein that can specifically bind to both soluble and membrane BAFF (20). Antagonist peptide has the advantages of simple synthesis and little toxic. Compared to Bellimumab, blisibimod has a higher affinity for BAFF (21). A phase I clinical trial confirmed the safety in SLE patients with moderate disease activities and explained that its pharmacological effect is by reducing naive B cells (22). In a phase III trial involving 442 patients with systemic lupus erythematosus disease activity index (SLEDAI) scores greater than 10 (23), there was no significant difference in remission between the blisibimod group and the placebo group. However, blisibimod significantly reduced the urinary protein/creatinine ratio and improved the serological index.

Atacicept is a humanized recombinant soluble fusion protein that contains the extracellular ligand binding domain of TACI, which is fused into the Fc portion of human IgG1, blocking both APRIL, and BLys (24, 25). In a phase Ib clinical trial, the safety, tolerability, and biological activity of atacicept were demonstrated in patients with mild to moderate SLE (26). A phase IIb study involving 306 SLE patients showed evidence of efficacy, particularly in patients with high levels of disease activity (27).

RC18, also called telitacicept, is a novel recombinant TACI-Fc fusion protein that can binding to BAFF and APRIL. As a dual-targeting drug, it can inhibit the two cytokines of BAFF and APRIL at the same time, more effectively reduce the immune response, and achieve the purpose of treating autoimmune diseases. According to the published data by RemeGen, 249 SLE patients were enrolled to evaluate the efficacy and safety of telitacicept in the treatment of moderate to severe SLE subjects. The results showed that there was a statistically significant difference in the clinical response rate (SLE responder index, SRI-4) between the telitacicept group (79.2%) and the placebo group (32%), which reached the primary endpoint of the clinical trial (NCT02885610). It is expected to be on the market in China in 2020 and has been approved for a phase II clinical trial by the FDA, with a phase III trial still in recruiting (NCT04082416).

### CD22/CD20 Inhibition

CD22 is a receptor on the surface of the B cell membrane and is initially expressed in naive B cells and also during the development of B cells; mature B cells have the highest CD22 expression, while plasma cells lack this surface molecule (28). CD22 can promote the proliferation and differentiation of B cells by regulating the signal transduction of the B cell receptor (BCR) (29). CD20 is a transmembrane calcium channel that is involved in the activation, proliferation and differentiation of B cells (30). CD20 exists in the late pre-B cells and goes through the maturation stage of B cells (31). Specifically, blocking these two B cell membrane surface receptors inhibits B cell proliferation and reduces the inflammatory response.

Epratuzumab is a humanized IgG1 mAb that targets CD22, which regulates B cell signals without a substantial reduction in the number of B cells (32). To date, seven clinical trials have examined the safety and efficacy of epratuzumab. Overall, these trials have demonstrated that epratuzumab is a well-tolerated drug with similar rates of adverse events, mainly infection and headache, in the placebo and epratuzumab groups (33). All tests showed an effect on B cells, and the number of B cells in peripheral blood decreased by 30–50%. Complement levels and autoantibody levels remained unchanged. Immunoglobulin levels stabilized, but data showed a 20% decrease in plasma IGM levels, which were not associated with infection (34–36).

Rituximab is a chimeric mAb with a human IgG1 domain and a mouse CD20 variable region (37). Rituximab is a classical B cell depletion therapy that has been approved for the treatment of RA. Although it failed trails in lupus nephritis (38), in a prospective observational study, 45/50 patients achieved complete remission (CR), or partial remission (PR) by a median time of 37 weeks (39). These results indicate that rituximab is still a promising therapy for the treatment of LN. A recent phase 2a, single-arm study involved 16 SLE patients with severe, refractory disease and they were treated with rituximab and belimumab. The responses are significant: 10/16 patients achieved low lupus disease activity, 11/16 reached renal responses. The combination therapy through complementary mechanisms, provides new insights in reducing the excessive autoreactive B lymphocytes (40). Another RCT of the combination of rituximab and belimumab is also under way (NCT03312907).

The complementary determinant region of veltuzumab is similar to that of rituximab. The binding activity and the effect on CDC were stronger than those of rituximab (41). Veltuzumab was effective in a patient with severe, drug-resistant SLE who did not respond to conventional treatment and was initially responsive to rituximab but subsequently deteriorated with high levels of anti-rituximab antibodies. After receiving veltuzumab treatment, the patient responded well, with decreased B cells and significantly improved clinical symptoms. Whether the application can be expanded is debatable (42).

### CXCR5/CXCL13 Inhibition

CXCR5, which is expressed in Tfh cells, mature B cells, and Treg cells, is involved in B cell migration and the formation of germinal cells (GCs) and guides disease-causing double negative (DN) T cells into lymphoid organs and kidneys (43). In CXCR5-deficient lupus murine model, the migration of DN T cell to lymph nodes was reduced and the kidney was not infiltrated (44). CXCL13, a ligand of CXCR5, is expressed in follicular dendritic cells and macrophages in secondary lymphoid organs (45). Both molecules play an important role in the maturation and migration of B cells.

Numerous studies demonstrate that circulating CXCL13 level in patients with SLE increases and may act as a novel target in the treatment of SLE (46). MAb5261 is a humanized IgG mAb against CXCL13 in preclinical stage (47). After the treatment of MAb5261, the number of germinal centers decreased and it interfered with the transport of B cells to the spleen in mice models of RA and multiple sclerosis. Its role in SLE needs to be studied.

## Targeting Costimulators

Immune activation of B cells requires the interaction of costimulatory signals with T cells, especially CD40/40L, CD28, Inducible T cell co-stimulator ligand (ICOSL), and CD80/CD86. Blocking this pathway indirectly inhibits the proliferation and activation of B cells and down-regulates autoantibody production, thus achieving a therapeutic effect (48, 49).

CD40 and CD40 ligand (CD40L) are a pair of costimulatory molecules. CD40L is mainly expressed in activated CD4+ cells and in monocytes, mast cells and basophils. After binding to CD40, which is expressed on the surface of B cells, CD40L regulates the interaction between CD4+ T cells and B cells, which is crucial for the activation, differentiation and memory generation of B cells (50–52). Dapirolizumab is an Fc peg-glycolated anti-CD40L antibody fragment (53). A phase I clinical trial that included 24 patients with SLE showed that the SRI-4 in the dapirolizumab group was obviously improved compared with that of the placebo group (5/12 vs 1/7) and the mechanism of gene expression changes was observed in blood RNA samples (54). A 24-week phase II trial is being recruited for (NCT02804763) to further study its efficiency in SLE.

Cytotoxic T lymphocyte associated protein 4 (CTLA4) is a receptor that is constitutively expressed in regulatory T cells and down-regulates the immune response when it binds to CD80 or CD86, which is expressed on the surface of antigen presenting cells (55). Abatacept is a recombinant protein composed of CTLA4 and immunoglobulin that binds to CD80/CD86 and inhibits the response pathway (56). Abatacept has been approved for arthritis and is currently being studied for SLE and lupus (57). A multicenter exploratory phase II clinical trial involving 175 SLE patients demonstrated its efficacy in SLE. The primary endpoint was the proportion of patients who deteriorated after steroid reduction began. After 12 months of follow-up, the rate of flares in the treatment group was 79.7%, and in the control group, it was 82.5%, which failed to reach the primary endpoint (58). However, given the pathogenesis of SLE, new clinical trials on abatacept should be designed to further confirm its potential use in SLE (57).

Inducible T cell co-stimulator ligand is highly expressed in CD4 and CD8 T cells in patients with SLE, leading to abnormal proliferation and activation of T cells and the generation of pathogenic autoantibodies (59). AMG557 is a mAb that binds to ICOSL. A phase Ib clinical trial showed its safety and potential curative effect (60). The phase II clinical trial of 112 patients showed that the KLH IgG reaction decreased significantly, but the KLH IgM reaction or IgG level had no obvious change. There were no significant changes in clinical features or other biological indicators (61).

CD80/CD86, a ligand of CD28 and CTLA4, plays a key role in autoimmune diseases and organ transplantation (62). There is no anti-CD80/CD86 antibody applied to clinical cases of SLE patients so far, but its application in follicular lymphoma has entered phase II clinical trials (63). Anti-CD86 (1D1) (64), a mAb that recognizes both human and mouse CD86, was used in the CGVHD-induced experimental lupus nephritis model. The data showed that blocking CD86 with 1D1 significantly alleviated proteinuria, autoantibody production, immune complex deposition, and renal parenchymal injury in mice.

## Targeting Cytokines

In the pathogenesis of SLE, many cytokines not only mediate the immune response but also serve as markers of disease progression, inhibiting the corresponding immune stimulation, and reducing the immune response (65, 66).

### IL-6 Inhibition

IL-6 is an important inflammatory factor that not only increases rapidly in the acute inflammatory response but also significantly up-regulates the immune response in immune diseases (67, 68). The increase in serum IL-6 levels is positively correlated with the disease activity of SLE (69). There is also a positive correlation with IL-17, which is why there have been studies on the bispecific antibody (BsAbs) of both factors.

Tocilizumab is a humanized anti-IL-6 receptor (IL-6R) mAb that blocks receptor binding to IL-6 (68). A phase I clinical trial involving 16 mild-to-moderate SLE patients studied the safety and efficacy of tocilizumab. The results showed that the level of resistant double-stranded DNA decreased by 47% and the disease activity significantly improved. However, tocilizumab resulted in a decrease in the absolute number of neutrophils, and the decrease was related to the dose of the drug, with 11 out of 16 patients becoming infected (70). The FDA has approved tocilizumab for the treatment of RA, but its use in SLE has not been well developed, given that it inhibits inflammatory responses and increases the risk of infection at the same time.

Sirukumab is a humanized mAb against IL-6 that neutralizes IL-6 in the blood and reduces inflammation (71). In a phase II clinical trial of lupus nephritis (72), the experimental group did not reach the expected endpoint, but urine protein decreased by 50% in 5/21 patients. More research need to be studied in lupus nephritis.

### IL-17 Inhibition

IL-17A, a member of the IL-17 family, is secreted mainly by Th17 cells. In SLE, IL-17A collectively recruits and activates neutrophils with other cytokines to amplify the inflammatory response, exacerbate inflammation and injury in targeted organs, and enhance the immune response (73).

Secukinumab, an anti-IL-17A mAb, has shown some promise in Phase II trials for multiple autoimmune diseases, particularly psoriasis (74). In a case report, a woman with psoriasis vulgaris that was complicated with refractory lupus nephritis was treated with secukinumab for elevated Th17 cells in her peripheral blood and substantial IL-17 infiltration in her renal interstitium, despite resistance to conventional treatment (75). After starting secukinumab treatment, the condition of this patient was improved. Further research needs to be performed in SLE.

### IFNα Inhibition

I interferon is a potent immune-stimulating factor produced by plasmacytoid DCs whose signaling pathway is mediated by type I interferon receptor (IFN R). In the pathogenesis of SLE, the activation of IFN system can be seen in most patients, manifesting an overexpression of type I IFN-regulated genes or an IFN signature (76). Blocking IFN suppresses the immune response and corrects the immune imbalance in SLE.

Sifalimumab is a humanized IgG1k mAb against IFN that is neutralized by binding to most subtypes of IFN (77). A phase IIb clinical trial that included 431 participants showed that only a group of patients with high levels of IFN SRI-4 significantly improved. Skin lupus erythematosus lesion area and severity index ——cutaneous lupus erythematosus disease area and severity index (CLASI) and joint count were significantly improved. No efficacy was found in reducing anti-dsDNA antibodies or improving C3/C4 levels, and subsequent exploratory analysis showed improvement in patients with low IFN expression (78). As with a recent multicenter phase II open-label study in Japan, the main adverse event was herpes zoster (78, 79). Although sifalimumab performed well in Phase II trial, its development was discontinued in favor of anifrolumab which had better results in phase II studies (80).

Rontalizumab is also a humanized IgG1 mAb against IFN, and clinical studies of rontalizumab have progressed to phase II. After observing 238 SLE patients for 24 weeks, it was found that although the primary and secondary endpoints were not reached, rontalizumab performed well in patients with low IFN signal measurements (ISMs), which was unexpected in terms of improving disease activity, reducing flares, and steroid reduction (81, 82). This is probably because of the difference in the mean trough concentrations of rontalizumab between the ISM-Low patients [56.5 (mu)g/mL] and ISM-High patients [39.4 (mu)g/mL], which may have contributed to the differential outcomes.

Anifrolumab is a humanized anti-IFNαR mAb that is effective in targeting IFNα (83). The first phase III trial of anifrolumab, TULIP-1, did not show significant influence at the primary endpoint according to SRI (84). But in TULIP -2, anifrolumab showed significant influence at the primary endpoint according to the BILAG-based combined lupus assessment (BICLA). The BICLA response rate of anifrolumab (48 weeks, 300 mg per 4 weeks) was 16.3 percentage points higher than placebo (47.8% and 31.5%, respectively) (85). This inconsistency in drug efficacy under different evaluation systems presents a challenge for the development of new drugs.

### TNFα Inhibition

TNF is an important inflammatory factor that mediates the autoimmune response. The level of TNF reflects the disease activity (DA) level of SLE and is positively correlated with the activity of lupus nephritis (86).

Infliximab is a humanized mAb against TNF that neutralizes TNF in peripheral blood. Patients with refractory lupus nephritis (87) have improved DA and proteinuria in response to infliximab. However, its safety and efficacy in treating SLE need further study.

### IL21/IL23 Inhibition

IL-21, a cytokine that is secreted by Th17 and Tfh cells, is highly expressed in the peripheral blood of SLE patients, induces the generation and differentiation of B cells and enhances the production of immunoglobulin (88). Up-regulation of IL-23 and its receptor has also been observed in lupus patients (89).

Ustekinumab is a mAb that acts on the IL12/IL23 subunit p40 is currently approved for use in psoriasis (90), with clinical trials in SLE underway. A phase II clinical trial involving 102 autoantibody-positive SLE patients who were receiving standard treatment showed (91) SRI-4 responses at 24 weeks in 37 (62%) of 60 ustekinumab patients and 14 (33%) of 42 placebo patients. The incidence of adverse events was higher in the ustekinumab group (78%) than in the placebo group (67%), with infection being the most common event.

## Targeting Complement

Complement mediates the deposition of immune complexes, which further lead to the involvement and damage of the deposition site, and blocking the complement-mediated pathway and reducing the immune response is a way to alleviate the involvement of SLE organs (92).

Eculizumab is a humanized anti-C5 mAb (93). It specifically binds to human terminal complement protein C5 and blocks the release of inflammatory factor C5a and the formation of C5b-9 by inhibiting the cleavage of human complement C5 to C5a and C5b. In several case reports (94–96), all lupus nephritis patients with eculizumab showed improved renal function and normal complement.

## Bispecific Antibodies in SLE

At present, there are around 100 BsAb drug candidates in clinical development (97), whereas only a dozen are associated with autoimmune disease (98). In SLE, in addition to two fusion proteins, atacicept and RC18, which are dual-target drugs, 5 BsAbs are in study (Table 2).

**TABLE 2:** Dual-target biological agents in SLE.

BsAb	Format	Targets Biological	Biological function	Stage in SLE to date	References
Atacicept	Fc fragment fusion	APRIL + BAFF	Inhibiting B cell maturation and survival	Phase IIb	(24–27)
RC18	Fc fragment fusion	APRIL + BAFF	Inhibiting B cell maturation and survival	Phase III	NCT04082416
Tibulizumab	IgG-scFv	BAFF + IL-17A	Inhibition of B cell maturation and inflammatory cytokines	Phase I	(100–102)
AMG570	IgG-scFv	ICOSL + BAFF	Inhibition of B cell maturation and T cell proliferation	Preclinical	(103)
22*-(20)-(20)	DNL-Fab	CD20 + CD22	Reducing B cells	Preclinical	(104, 105)
Obexelimab	Fc mutated IgG	CD19 + FcγRIIb	Suppressing innate and adaptive B cell activation	Phase II	(106–109)
MT-6194	IgG-Fynomer	IL-17A + IL-6R	Inhibiting inflammation	Preclinical	(110, 111)

B cell activation factor is a critical target for these pending BsAbs, with more than half of the drugs designed to be targeted at it. This is due not only to its important role in the pathogenesis of SLE, but also to the confidence generated by Belimumab’s successful development (99). In addition, how to design cytokines into the network of dual targets is also a problem with research value.

**FIGURE 2:**
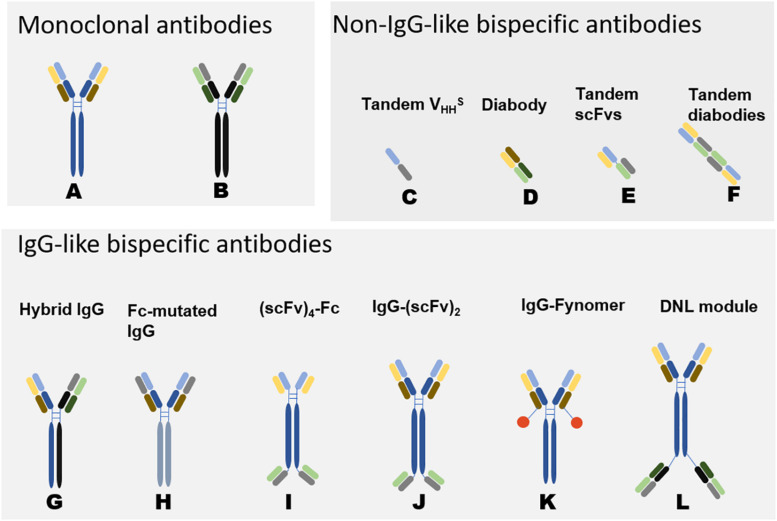
Schematic Diagram of Bispecific Antibodies (118). Natural antibodies are tetramers of two light chains (L) and two heavy chains (H) and contain two identical Fab domains with binding antigen sites and one Fc domain. Bispecific antibodies can be divided into two categories according to the presence or absence of Fc segments: non-IgG-like BsAbs and IgG-like BsAbs. Non-IgG-like BsAbs have low molecular weights and robust tissue penetration and exert effects through specific structural domains bound to antigens. However, due to their small molecular weights and lack of receptor-binding Fc structure, the antibodies cannot mediate antibody-dependent cell-mediated cytotoxicity (ADCC) and complement-dependent cytotoxicity (CDC) (118, 119). Because of random assembly of the different chains, the design of IgG-like BsAbs mainly focuses on how to solve the mismatch between two different heavy chains and the mismatch between heavy chains and light chains. IgG-like BsAbs have improved stability but strong immunogenicity. The development of genetic engineering technology has promoted the preparation of IgG-like BsAbs (120). **(A, B)** represent two different monoclonal antibodies. **(C–L)** are variants of **(A, B)**, representing some common structures of bispecific antibodies. The origin of the light and heavy chains can be determined by their colors. The corresponding format name is marked above the antibody, different formats have their own characteristics in manufacturing and effect functions (30). DNL: In Dock-and-lock (DNL) method, antibody fragments are fused to heterodimerizing proteins.

Tibulizumab is a novel BsAb that is composed of two divalent antibodies that act independently and targets both BAFF and IL-17A (100) (Figure 2J). BAFF is not only involved in the activation of B cells but also promotes the proliferation of Th17 cells, thereby mediating the downstream immune response. IL-17, which is secreted by Th17 cells, in turn promotes inflammation (101). Blocking both IL-17 and BAFF has advantages that anti-17 mAbs and anti-BAFF mAbs alone cannot achieve (102). Tibulizumab effectively antagonizes BAFF and IL-17 in both cellular and live mouse models. In the Cynomolgus monkey model, the development and survival of B cells were inhibited, the circulatory function was complete, and the half-life was prolonged (100). A phase I clinical trial is currently ongoing to study the safety, tolerability, pharmacokinetics and pharmacodynamics of tibulizumab in Sjogren’s syndrome.

AMG570 is a BsAb that targets ICOSL and BAFF for the treatment of autoimmune diseases such as SLE (103) (Figure 2J). The current research on AMG570 is still in the preclinical stage. Treatment with ICOSL/BAFF BsAb or combination therapy was more efficacious than that of a single ICOSL or BAFF inhibitor in a mouse lupus model. Dual ICOSL and BAFF inhibition was also effective in the mouse collagen-induced arthritis (CIA) model. In cynomolgus monkeys, B cells were reduced significantly after treatment with AMG570.

22^∗^-(20)-(20) is a bispecific hexadecavalent antibody (bsHexAb) that targets CD20 and CD22 (104). It is composed of the Fc of epratuzumab and four Fabs of veltuzumab, and a CD20-targeting immunocytokine, using the Dock-and-Lock (DNL) method (Figure 2L). This method combines recombinant engineering with site-specific conjugation, allowing the construction of various complex, yet defined, biostructures with multivalency and multispecificity (105). *In vitro* experiment, the 22^∗^-(20)-(20) mediates a broad and potent trogocytosis of multiple B-cell surface proteins with only moderate B-cell depletion compared to veltuzumab (104).

Obexelimab (XmAb5871) is a humanized Fc-engineered antibody that binds to CD19 on the B cell surface and has a better affinity for Fcγ receptor IIb (FcγRIIb) to inhibit the function and activation of B cells (106–108) (Figure 2H). CD19 is expressed in almost all stages of B cells, because of its wide expression, the use of therapeutic antibodies against CD19 in SLE is limited (109). In a phase II clinical trial involving 104 SLE patients, obexelimab showed some inhibition of disease activity. SLEDAI scores increased by no more than 4 points in 42% of patients in the treatment group compared with 23% in the placebo group.

MT-6194 is a bispecific antibody that targets both IL-17A and IL-6R using a gene fusion technique that combines the anti-IL-17A Fynomer 11L9C09 with anti-IL-6R tocilizumab light chain C-terminus (Figure 2K). Fynomer is a small protein, but it does not act as a drug on its own. Instead, it forms a fusion protein with an intact antibody molecule, allowing the complex to bind to two different targets simultaneously (110). Currently in preclinical studies, MT-6194 inhibits inflammation better than each cytokine alone in a mouse model of delayed hypersensitivity inflammation (111).

## Bispecific Antibodies and Their Application in Other Diseases

Bispecific antibodies have been in development for some time. In 2014, blinatumomab (CD19 and CD3) became the first FDA-approved BsAb for the treatment of lymphoblastic leukemia (112). Emicizumab (Factor IX and Factor X), for the treatment of hemophilia, was marketed in 2017, becoming the first BsAb for a noncancer disease (113). Although BsAbs have been studied in various fields, such as infectious diseases, diabetes, and autoimmune diseases, their development is still in the early stage.

For autoimmune diseases, the pathogenesis involves complex immune abnormalities, involving multiple cytokines. Theoretically, this should be the ideal application of BsAbs, but in practice, it presents great challenges, mainly due to the following limitations. First, autoimmune diseases have very strong heterogeneity. The specific cytokines and cell levels in different patients vary greatly, which limits the clinical application of the corresponding antibody. Although these problems also exist with mAbs (Figures 2A,B), because monoclonal antibodies involve only one site and BsAbs involve two, BsAbs are more restricted in their application to the immune network. Second, immunogenicity limits the use of BsAbs. BsAbs are mostly fragment-based and nonnative formats, which may have stronger immunogenicity than simple IgG (114). Patients with autoimmune diseases have an overly strong immune response, which may produce anti-antibodies and crossreact with the use of biological products. At the same time, the immune complex formed by the double-targeting effect of BsAbs may be too large, and additional damage may be caused if deposition occurs. Third, there are many kinds of structures of BsAbs (Figures 2C–L), how to choose the most suitable form according to the needs of the target is a problem that needs to be studied.

Although there are some myths about the use and development of BsAbs, they also have obvious advantages. The first is the increase in the number of mechanisms of action (115), which can simultaneously target multiple activation pathways and more robustly inhibit immune responses. The second is that BsAbs are a special antibody mixture, and the ratio of the two antibodies is been determined at the very beginning. Therefore, it is possible to determine the safe dose, maximum dose and other issues during preliminary clinical trials. It is not necessary to consider the dose and effect of the two when using monoclonal antibodies in combination. The future of BsAbs is precision medicine. Once the production cost is greatly reduced and the research and development technology is fully mature, BsAbs and multiple antibodies can be customized according to the specific situation of each subtype or even each patient to achieve the relief of patients’ symptoms.

## Conclusion

Biological therapies for SLE are diverse, covering all B cell-associated processes, from proliferation and differentiation to activation. There are some agents that work well, such as belimumab, rituximab and atacicept, on the market. RC18 is expected to be the world’s first dual-target biological drug for SLE. However, most of the biological agents are still in the phase II and III clinical stages or even in the preclinical stage, and have poor efficacy, side effects and other issues. In addition, many agents that have been widely used in other diseases are gradually broadening their indications and are being tested in SLE, but their efficacy needs further verification.

At present, biological agents are mainly used for patients with moderate and severe SLE. In the case that immunosuppressive agents and hormone therapy are ineffective, biological agents are used to control the disease (116). Therefore, there is still a question of whether the combination of drugs is reasonable. Rotalizumab is a good example for us to pay attention to the subgroup patients and give personalized treatment, according to the corresponding biological response. BsAbs are also currently being studied in SLE. The advantage of BsAbs is that blocking multiple activation pathways not only reduces the immune response but also changes the existing market (117, 118). Certainly, BsAbs against SLE are still in a relatively preliminary stage, and the specific dose problems need further clinical trials to be determined. Both mAbs and BsAbs have the problem of producing anti-antibodies, which leads to tolerance. Therefore, the use of therapeutic antibodies for *in vitro* immunosorbent therapy is also a promising application. Ustekinumab is also an insightful idea. Combined with the subunit of IL21/IL23, ustekinumab can affect the two up-regulated pathways. Finding more specific key targets is critical in the development of antibodies.

## Author Contributions

BY wrote the manuscript. MZ did the editing. HW and QL revised the manuscript. All authors contributed to the article and approved the submitted version.

## Conflict of Interest

The authors declare that the research was conducted in the absence of any commercial or financial relationships that could be construed as a potential conflict of interest.
